# Electromyography (EMG) Analysis of Multi-Regional Lower Extremity and Trunk Musculature During Sidelying Hip Abduction With Frontal Plane Stabilization

**DOI:** 10.7759/cureus.43523

**Published:** 2023-08-15

**Authors:** Corbin Hedt, Bradley Lambert, Jordan A Jackson, Erika Brager, Grace Forbes, Jordan Ankersen, Patrick McCulloch

**Affiliations:** 1 Orthopedics & Sports Medicine, Houston Methodist Hospital, Houston, USA; 2 Physical Therapy, True Sports Physical Therapy, Timonium, USA; 3 Kinesiology, Rice University, Houston, USA

**Keywords:** quadratus lumborum, gluteus maximus, compensatory patterns, lower extremity, knee, hip, emg, hip abduction, gluteus medius, exercise

## Abstract

Sidelying hip abduction (SHA) is a common exercise utilized in rehabilitation to strengthen the gluteus medius (GMed). Alterations in the exercise can produce different patterns of muscular activity. No studies have examined the effect of mechanical pelvic stabilization during SHA. This study enrolled 19 participants (male = 11, female = 8) who performed the same SHA exercise under two randomized conditions: standard and with a mechanical block to prevent frontal-plane movement. Electromyographic amplitudes during exercise were obtained through surface electrodes and compared against maximum voluntary isometric contraction (MVIC) testing: GMed, gluteus maximus, biceps femoris, tensor fascia latae, quadratus lumborum, and vastus lateralis. While no significant differences were found in GMed activity during SHA with or without pelvic stabilization, reduced concomitant activation of other musculature was observed, potentially producing a more isolated exercise for the GMed with less compensatory activity.

## Introduction

Sidelying hip abduction (SHA) is a common and effective exercise used clinically to target the gluteus medius (GMed) muscle [1-4]. When comparing the different methods of exercise involving hip abduction, there seems to be a clear variance in GMed activation, potentially altering the effectiveness of an exercise based on position, alignment, or accessory muscle recruitment [1,2,5-9]. Contrasted with exercises of similar biomechanical demand, SHA promotes greater isolated GMed activity through a simplified manner, making it a common choice for those undergoing rehabilitation of the hip or lower extremities [4,5]. Investigating alterations of SHA may provide insight into effective and clinically-feasible exercises that can successfully target and strengthen the GMed. However, currently unknown are how positional or biomechanical variations of the SHA exercise alter the activity patterns of the GMed and other lumbopelvic musculature.

The GMed is a flat, triangular muscle that functions primarily as a hip abductor [10]. It consists of three segments; its anterior and middle segments are located superficial to the gluteus minimus with the posterior segment just deep to the gluteus maximus (GMax) [10]. The GMed also acts as an important pelvic stabilizer against gravity in both frontal and transverse plane activities, such as running, walking, or in a unilateral stance [8,10]. GMed dysfunction or weakness can manifest through functional hip impairments and the adoption of compensatory patterns such as excessive quadratus lumborum (QL) activity [8]. Furthermore, GMed weakness and subsequent altered kinematics at the lumbar spine, hip, or knee regions have been linked to several lower-extremity conditions such as patellofemoral syndrome, iliotibial band syndrome, chronic ankle instability, and anterior cruciate ligament injuries [11]. Therefore, strengthening the GMed muscle is often a primary goal in rehabilitation or injury prevention of lower extremity or lumbar injuries [12,13].

Clinicians and physical therapists will often monitor movement quality when prescribing therapeutic exercises to ensure appropriate muscle targeting and minimize unwanted variations. Several studies have attempted to examine variants in SHA with alterations in position, angle, or resistance [1,2,5,8,14,15]. Lee et al. concluded that a minor lateral rotation with hip abduction increased GMed involvement with reduced tensor fascia latae (TFL) activity [5]. By diminishing compensatory contributions of accessory muscles, therapists can confidently prescribe exercises that closely target the GMed by better isolating the frontal plane action of the muscle. While current research has provided meaningful information on transverse and sagittal plane variability [15,16], no studies have examined GMed activity while attempting to minimize frontal plane contributions by stabilizing the pelvis. By understanding the recruitment patterns of regional musculature including the GMed with these controlled modifications, clinicians and patients may be empowered with a more meaningful exercise response. Therefore, the purpose of this study was to determine if the addition of a pelvic stabilizer to limit frontal plane movement produces altered lower extremity or trunk muscle activity during SHA. The authors hypothesized that subjects would achieve higher GMed activity relative to all other musculature with frontal-plane pelvic movement controlled, especially during higher repetitions. The findings of this study will provide valuable information for clinicians to assist in exercise prescription to strengthen the GMed. 

## Materials and methods

Institutional Review Board of Houston Methodist Research Institute approved the study (approval number: PRO00029919). A total of 19 healthy subjects were included in the study. Participants with a recent history of spine or lower extremity injury, limb amputation, or any other known contraindications (physician-ordered) to exercise were excluded. All participants performed the same SHA exercise on a firm, padded therapy table. 

**Figure 1:**
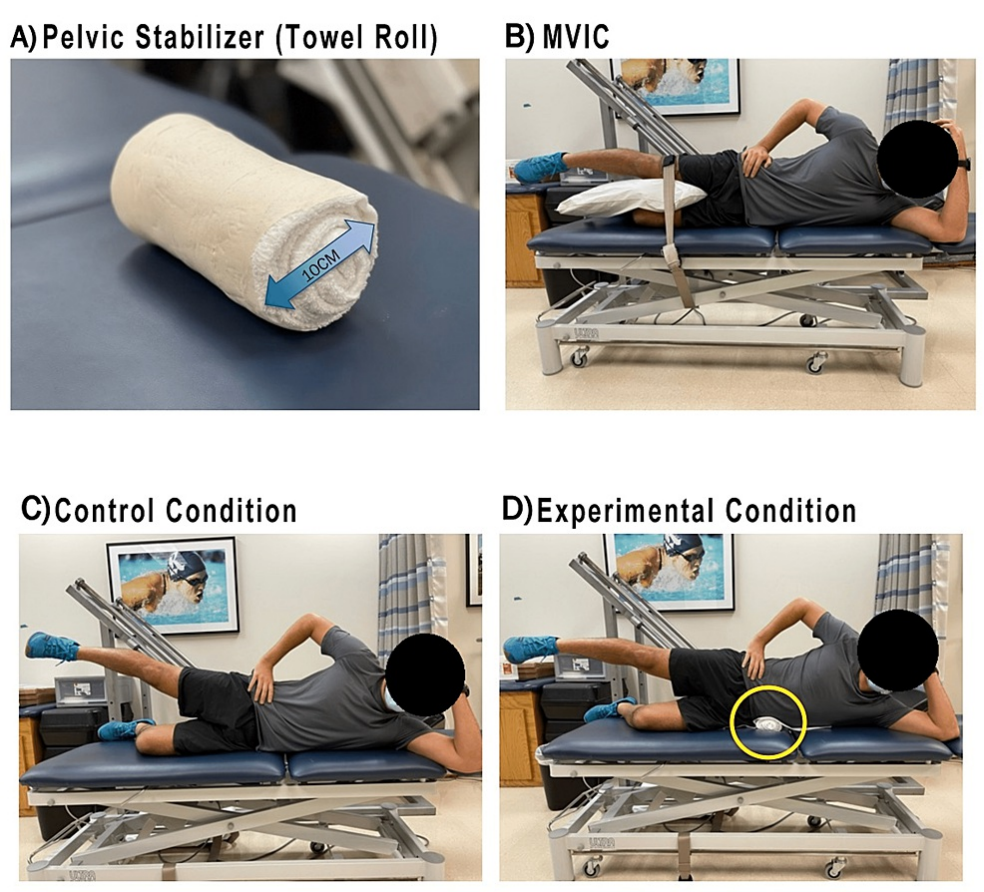
Experimental set-up MVIC: maximum voluntary isometric contraction

Participants were allowed to choose their preferred limb for exercise. The exercise was performed by all participants under two conditions: experimental and control. The experimental condition utilized a firm 10 cm towel roll that was covered in clean, sanitary plastic (Figure 1A). This was used as a mechanical block and placed under the subject at the level of the iliac crest while they were in the sidelying position (Figure 1D). The towel roll was not utilized in the control condition (Figure 1C). The order of each condition was randomized prior to the exercise sessions.

Before exercising under either condition, all subjects were marked with surface electrodes (Delsys Incorporated, Natick, Massachusetts, United States) in a similar fashion based on palpation from the same, skilled physical therapist for the following musculature: GMed, GMax, vastus lateralis (VL), QL, biceps femoris (BF), and TFL. The methods for electromyography (EMG) recording and analysis have been previously published [17,18]. Briefly, after skin preparation using isopropyl alcohol and confirmation of wireless connectivity, EMG was recorded during the acquisition of maximal voluntary isometric contraction (MVIC) for each muscle as well as the experimental trials at a sampling rate of 1926 samples per second with a filter range of 20-450 Hz as specified by the manufacturer. The MVIC was obtained with the subject in the same position as the SHA exercise, at least five minutes prior to either condition, with a stable belt wrapped around the testing table and just proximal to the lateral femoral condyle. This method has been previously utilized in earlier studies [1,5,15,16].

Subjects initiated the exercise when indicated by the investigator, using a metronome to normalize repetition speed and ensure good movement quality. Under both conditions, the subject performed 40 continuous repetitions at a rate of 20 repetitions per minute. There was a standardized rest break of at least five minutes in between each exercise to allow for optimal recovery. EMG sampling was analyzed following each participant’s successful exercise session. Mean EMG was assessed for each contraction, averaged across five repetition intervals, for the first 20 repetitions, and for the last 20 repetitions of exercise for each muscle. Next, the raw EMG signal then underwent root mean square transformation and normalization to percent MVIC prior to final analysis, as is common with similarly published works [17,18].

Statistical analysis

All analyses were performed using IBM SPSS Statistics for Windows, Version 24.0 (2016; IBM Corp., Armonk, New York, United States). Based on previous EMG-based investigations utilizing similar repeated measures designs within a single group of participants under different stimulus conditions [17,18], for a power of 0.80 (α=0.05) and a minimum detectable difference of 10% in relative EMG amplitude between conditions at the same matched measurement timepoint, a minimum of 15 participants was deemed to be required. Therefore, to account for potential dropouts, 20 participants were recruited for this investigation with a single dropout due to scheduling conflicts resulting in 19 participants that completed the investigation. 

A paired-sample t-test was used to compare EMG amplitude (EMGa) at each of the averaged contraction intervals, averaged across the first 20 contractions, and averaged across contractions 21-40. Type-I error was set at =0.05. For all significant pairwise comparisons, effect size (ES) was calculated using a Cohen’s d statistic and interpreted as follows: <0.1 (Negligible, N); 0.1-0.3 (Small, S); 0.3-0.5 (Moderate, M); 0.5-0.7 (Large, L); >0.7 (Very Large, VL).

## Results

A total of 19 participants were included in the study. The demographical characteristics of the participants are given in Table 1.

**Table 1:** Demographical characteristics of the participants values are given as mean (±SD)

Male (n = 11)			Female (n = 8)		
Age (years)	Height (cm)	Weight (kg)	Age (years)	Height (cm)	Weight (kg)
28.4 (±4.3)	180.3 (±6.4)	81.6 (±7.2)	27.5 (±1.9)	164.1 (±9.4)	61.2 (±6.2)

**Figure 2:**
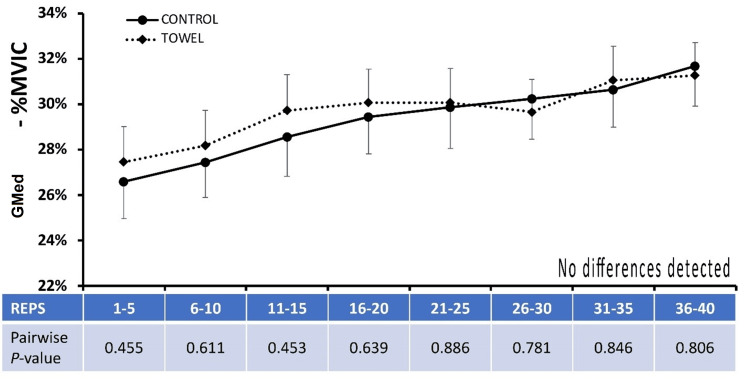
Gluteus medius EMG amplitude Data are presented as means ± 95%CI for mean EMG amplitude averaged across five contraction intervals and normalized to maximal voluntary isometric contraction (%MVIC) for the GMed muscle under the Control and Experimental (Towel) conditions.  Mean %Difference ± 95%CI between Experimental (Towel) and Control conditions is also provided.  P-values for pairwise comparisons at each matched contraction interval are also presented.  No differences between conditions were detected at P<0.05. EMG: electromyography; GMed: gluteus medius

**Figure 3:**
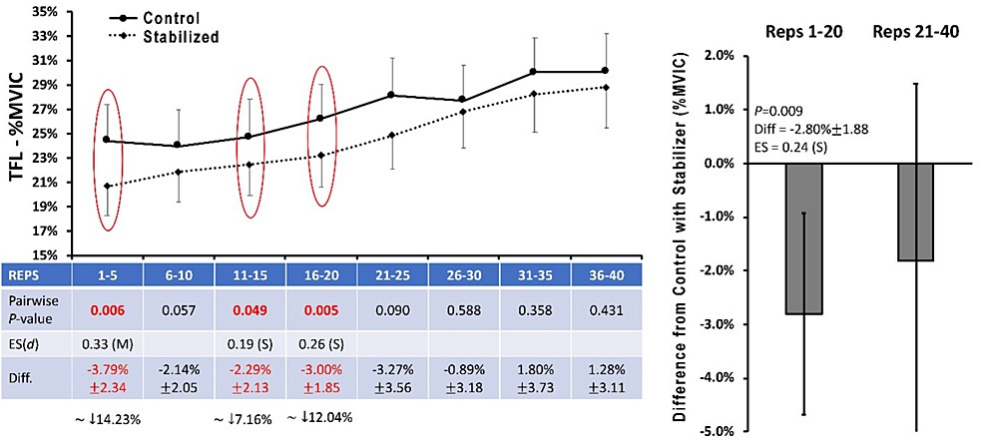
Tensor fascia latae EMG amplitude Data are presented as means ± 95%CI for mean EMG amplitude averaged across five contraction intervals and normalized to maximal voluntary isometric contraction (%MVIC) for the TFL muscle under the Control and Stabilized (with towel) conditions.  Mean %Difference ± 95%CI between Stabilized (with towel) and Control conditions is also provided.  Data are also presented as the "difference from Control with Stabilizer" averaged across repetitions 1-20 and 21-40.  P-values for pairwise comparisons at each matched contraction interval are also presented.  For comparisons reaching statistical significance at P<0.05, ESs are provided as a Cohen's d statistic and interpreted as follows:  0-0.1, Negligible (N); 0.1-0.3, Small, (S); 0.3-0.5, Moderate (M); 0.5-0.7, Large (L); >0.7, Very Large (VL). TFL: tensor fascia latae; EMG: electromyography; ES: effect size

**Figure 4:**
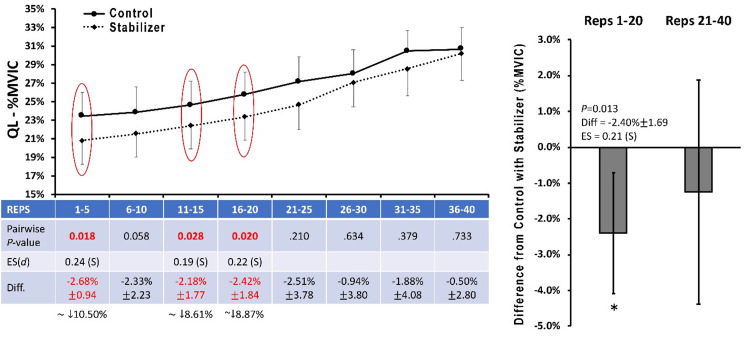
Quadratus lumborum EMG amplitude Data are presented as means ± 95%CI for mean EMG amplitude averaged across five contraction intervals and normalized to maximal voluntary isometric contraction (%MVIC) for the QL muscle under the Control and Stabilized (with towel) conditions.  Mean %Difference ± 95%CI between Stabilized (with towel) and Control conditions is also provided. Data are also presented as "difference from control with stabilizer" averaged across repetitions 1-20 and 21-40.  P-values for pairwise comparisons at each matched contraction interval are also presented.  For comparisons reaching statistical significance at P<0.05, ES are provided as a Cohen's d statistic and interpreted as follows:  0-0.1, Negligible (N); 0.1-0.3, Small, (S); 0.3-0.5, Moderate (M); 0.5-0.7, Large (L); >0.7, Very Large (VL). QL: quadratus lumborum; EMG: electromyography; ES: effect size

There were no significant differences observed between conditions for mean EMGa within the GMed (Figure 2), GMax, or BF between control and stabilization states. Modest, but significant, reductions of mean EMGa were found for the TFL (Figure 3) and QL (Figure 4) for repetition intervals 1-5, 11-15, 16-20, and overall for the first 20 repetitions when SHA was performed with a pelvic stabilizer compared to the control condition (P<0.05).

**Figure 5:**
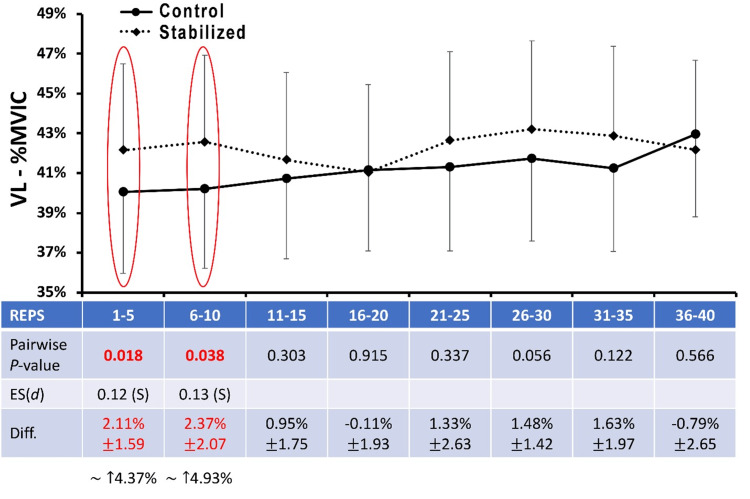
Vastus lateralis EMG amplitude Data are presented as means ± 95%CI for mean EMG amplitude averaged across five contraction intervals and normalized to maximal voluntary isometric contraction (%MVIC) for the VL muscle under the Control and Stabilized (with towel) conditions.  Mean %Difference ± 95%CI between Stabilized (with towel) and Control conditions is also provided.  P-values for pairwise comparisons at each matched contraction interval are also presented.  For comparisons reaching statistical significance at P<0.05, ES are provided as a Cohen's d statistic and interpreted as follows:  0-0.1, Negligible (N); 0.1-0.3, Small, (S); 0.3-0.5, Moderate (M); 0.5-0.7, Large (L); >0.7, Very Large (VL). VL: vastus lateralis; EMG: electromyography; ES: effect size

Furthermore, analysis of the VL (Figure 5) revealed a small, but significant, elevation in mean EMGa for repetition intervals 1-5 and 6-10 while utilizing the pelvic stabilizer (P<0.05) compared to the control condition. There were no significant findings related to peak EMG activity.

## Discussion

The purpose of the present investigation was to examine lower extremity and trunk musculature during the SHA exercise with and without a pelvic stabilizer to limit frontal plane movement. The authors hypothesized that the observed muscle groups will differ in EMGa with a stabilizer to favorably increase GMed activity relative to surrounding structures.

While the present findings indicate no difference between conditions for GMed activity during SHA, reductions in concomitant activation of other hip stabilizer muscles (TFL and QL) indicate that the use of pelvic stabilization via a towel block may assist in reduced compensatory activity and improved isolation of the GMed during hip abduction. Further longitudinal trials are required to determine the effect of using pelvic stabilizers over time during rehabilitation or preventative training; however, the present findings provide initial support for clinical application.

Regarding the primary muscle of interest, the addition of a pelvic stabilizer does not necessarily equate to heightened utilization of the GMed itself. However, reduced EMGa in surrounding muscles with the frontal plane block may provide the potential to reduce unfavorable compensations which are likely present within pathologic populations. These results are in contrast to a similar study by Kyung-Mi et al., who concluded that pelvic stabilization during SHA did increase GMed activity, although through a different means of mechanical movement reduction with compression [16]. Therefore, if there is a specific intent to maximize GMed utilization during SHA, other methods of stabilization could be preferable to the towel roll used in this study.

While direct GMed strengthening is often the focus of rehabilitation professionals and physical therapists, occasionally therapeutic activities will encapsulate more of a neuromuscular control focus to improve movement quality and reduce variability in functional tasks such as ambulation, stair climbing, squatting, and recreational/sports demands [13,19-22]. The authors in this investigation anticipated that the use of a pelvic stabilizer when performing hip abduction would minimize any pelvic hiking in the frontal plane, a common compensatory pattern observed in GMed weakness, dysfunction, or fatigue [23-25]. Interestingly, the data presented in this work seems to corroborate a reduction in accessory activity, namely in the QL and TFL musculature. Previous studies have demonstrated that the QL is highly active in an effort to produce a “hip hike”, especially during functional weight-bearing activities including gait [12,23,24]. This action allows for the contralateral limb to swing through as a means of compensating for poor gait quality due to GMed weakness or lumbar/lower extremity pathology [12,23,25]. In addition to their findings of higher GMed activity, Kyung-Mi et al. also found a reduction in QL utilization during SHA with stabilization via a compression belt [16]. While the pelvic stabilizer did not result in heightened GMed activity, the reduction in QL activation may be beneficial in reducing unwanted hiking at the pelvis to improve functional movement patterns. It is important to note that the use of a towel roll to achieve pelvic stabilization may be more clinically feasible or economical, limiting the need to purchase and obtain further equipment.

Moreover, other studies have examined the effects of exercise modification in GMed and TFL activity as preferential recruitment of the TFL has been observed in GMed weakness and hip pathology [3,9,26,27]. Wilcox and Burden demonstrated significant variability in GMed activity with the clamshell exercise, but with very little TFL activity regardless of position [28]. Selkowitz et al. examined several other hip abduction exercises to determine which produced the greatest gluteal recruitment with minimal TFL activity [3]. Interestingly, the SHA exercise, which is ranked as one of the higher ratios of MVIC% for TFL to GMed activation, ranked as the highest for outright GMed utilization [3]. Therefore, the utility of the SHA exercise seems to be favorable for the GMed, and the data presented in this investigation may help to improve the exercise by reducing compensatory TFL involvement.

Notably, the VL experienced elevated activation while under the stabilized condition compared to the control for the first 10 contractions (Figure 5). To date, no studies have specifically investigated this phenomenon. Previously published data indicate that VL involvement with SHA is typically nominal compared to other exercises such as lunging, squatting, or stepping [29]. The authors hypothesize that the pelvis stabilizer lends to the potential for a better length-tension relationship and elevated EMGa of the VL at the onset of exercise, similar to the principles observed in the early research on modifying muscle length during strengthening [30]. Future research may help to elucidate the role of the VL or other quadriceps muscles to a greater degree during SHA.

Next, other musculature (GMax and BF) observed during this study did not provide significant differences between conditions. While the SHA exercise seems to be a valuable tool to successfully address GMed insufficiency, currently available data indicate that it may not be particularly impactful for the GMax or hamstring musculature compared to higher-level weight-bearing exercises that specifically target those muscle groups [1,29]. Boren et al. propose that higher MVIC% for the GMax tends to arise from higher-level weight-bearing exercises such as single-leg squats and deadlifts [1]. In agreement with their data, Muyor et al. also indicate that single-leg squats and lunges garner the greatest GMax activity [29]. While also producing a high degree of EMGa for the GMed, unfortunately, these activities may not be appropriate for populations with reduced strength, motor control, or hip pathology/pain.

Limitations

The present study is not without limitations, which should be considered. Of note, all individuals that were recruited for this study were healthy without a pathologic lumbar or hip condition, which could potentially alter muscle activity patterns compared to patient populations with pain or movement limitations. Therefore, the present data can only be generalizable to healthy adults until further investigations are performed on pathologic populations undergoing rehabilitation. Secondly, we do not discount that imposing a mechanical stabilizer may induce discomfort with exercise based on the implement utilized, subject positioning, or the surface on which they are performing the exercise and should be further studied in various populations. Finally, the sidelying positioning and electrode placement utilized for all subjects may contribute to variance between participants; however, the same investigator performed the electrode and exercise setup throughout the entirety of the study. All subjects did perform both conditions (randomized) within the same session to minimize electrode variability.

Future directions

The results of this experiment warrant further research on methods to increase GMed EMGa while mitigating compensatory muscle activation. We propose investigating the effect on GMed EMG activity through modification of SHA via positional changes or the added effect of resistance. Additionally, investigation of the incorporation of a frontal plane stabilizer of the pelvis with exercises other than SHA may result in a greater GMed activation or a reduced EMG activity in the surrounding musculature. Finally, research into the muscular contributions during SHA should be replicated in pathologic populations.

## Conclusions

The EMGa data sampled within this investigation revealed no significant difference in GMed activity during sidelying abduction with or without a pelvic stabilizer for the frontal plane. However, the use of the stabilizer does reduce concomitant activation of certain musculature prone to be maladaptive to optimal function. Thus, the addition of the pelvic stabilizer to SHA may assist in decreasing compensational activation of pelvic stabilizers in a clinical setting while maintaining good levels of GMed activity.
